# Supplementary material for: Pre-Diagnostic Circulating Resistin Concentrations Are Not Associated with Colorectal Cancer Risk in the European Prospective Investigation into Cancer and Nutrition Study
Source: Cancers (Basel). 2022 Nov 9;14(22):5499. doi: 10.3390/cancers14225499 (PMC9688251; doi:10.3390/cancers14225499)
Supplement: Supplementary file 1 [file cancers-14-05499-s001.zip › cancers-1953682-supplementary.pdf]

Title: Pre-Diagnostic Circulating Resistin Concentrations Are Not Associated with Colorectal Cancer Risk in the European Prospective Investigation into Cancer and Nutrition Study

Supplementary Materials

Figure S1: Association of pre-diagnostic resistin concentrations and risk of CRC.

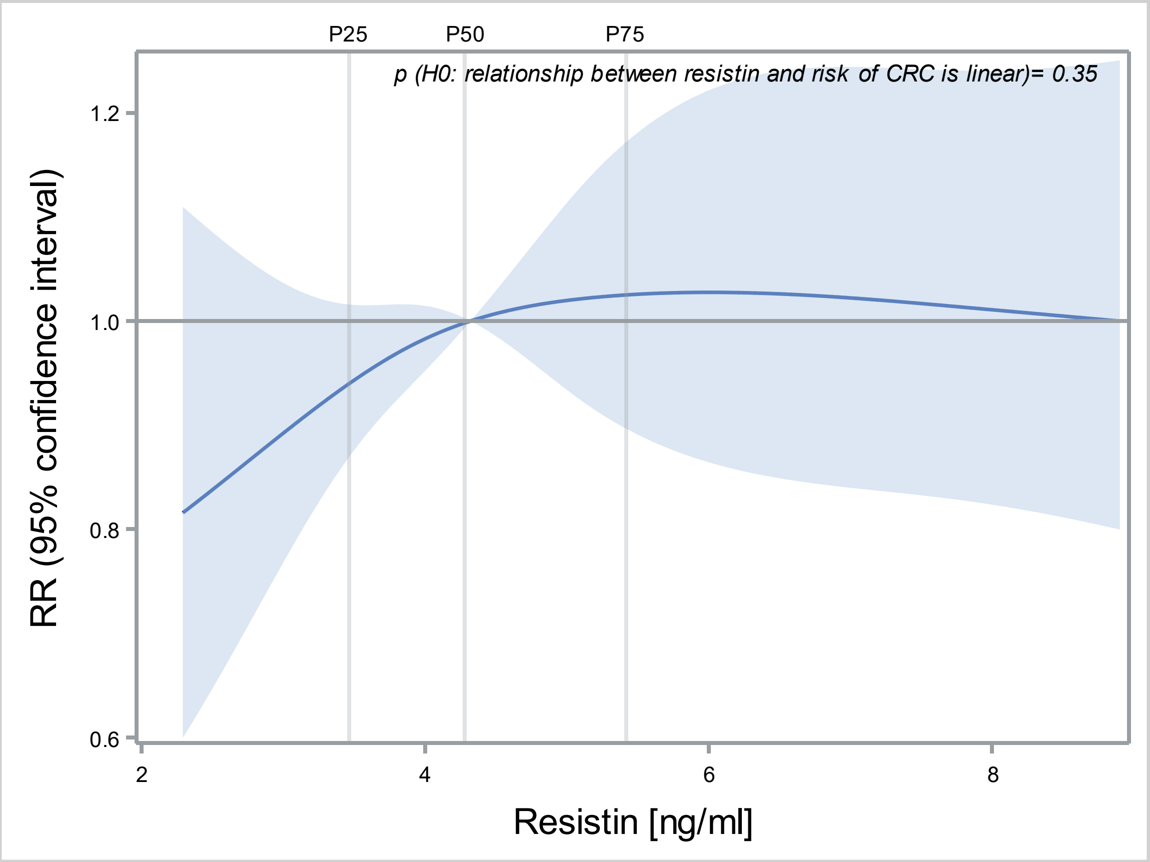

Association of circulating resistin concentrations with colorectal cancer in a spline regression model based on a conditional logistic regression with restricted cubic splines plotted with four knots at the 5th, 35th, 65th, and 95th percentiles of resistin concentration distribution. Solid lines indicate the relative risk, and shaded blue areas indicate the 95% confidence intervals. The reference of the spline plots is the median of resistin concentrations (4.32 ng/ml). Grey vertical lines indicate the 25th (P25), 50th (P50), and 75th (P75) percentiles of resistin concentrations.

All models were conditioned on matching factors including age, sex, study center, time of the day at blood collection, and fasting status; women were further matched by menopausal status, phase of the menstrual cycle at blood collection, and use of oral contraceptives at blood collection; postmenopausal women were matched by hormone replacement therapy use. P-values for non-linearity were derived from a likelihood ratio (LR) test to compare full multivariable-adjusted conditional logistic regression models including both the linear and cubic spline term and reduced multivariable-adjusted conditional logistic regression models with only the linear term.

Table S1. Characteristics of 1293 colorectal cancer cases and the 1293 matched controls in the nested case-control study, European Prospective Investigation into Cancer and Nutrition, 1992–2005

	Colorectal cancer cases (n=1293)	Matched controls (n=1293)
Colorectal cancer subtype, n (%)		
Colon cancer	757 (58.6)	—
Rectal cancer	475 (36.7)	—
Unspecified	61 (4.7)	—
Age at blood collection, years, mean (SD) [a]	58.1 (7.0)	58.1 (7.0)
Women, n (%) [a]	681 (52.7)	681 (52.7)
Postmenopausal women, baseline, n (%)[a]	505 (39.1)	515 (39.8)
Fasting (>6 h), n (%) [a]	350 (27.1)	350 (27.1)
BMI, kg/m2, mean (SD)	26.7 (4.1)	26.3 (3.8)
Waist circumference, cm, mean (SD) [b]	90.4 (12.8)	88.5 (12.3)
Diagnosed diabetes at baseline (self-reported or Hba1C >= 6.5%), n (%)	106 (8.2)	64 (5.0)
Current smoker, n (%)	328 (25.4)	323 (25.0)
University or higher education level, n (%)	221 (17.1)	229 (17.7)
Physically active (moderately active, or active), sex-specific n(%)	262 (20.3)	319 (24.7)
Alcohol abstainers (under 0.3 g/day), n(%)	117 (13.7)	189 (14.6)
Alcohol consumption, g/day, median (Q1–Q3) [b]	8.7 (1.5 - 24.1)	8.0 (1.7 - 21.1)
Energy intake, Kcal/d, median (Q1–Q3) [b]	2074 (1682 - 2498)	2029 (1639 - 2464)
Red meat, g/day, median (Q1–Q3) [b]	48.1 (24.6 - 76.2)	46.5 (24.8 - 74.1)
Processed meat, g/day, median (Q1–Q3) [b]	25.7 (13.7 - 44.5)	25.2 (13.3 - 44.4)
Dietary fiber, g/day, median (Q1–Q3) [b]	21.9 (17.5 - 27.3)	22.9 (18.0 - 27.7)
Fruit intake, g/day, median (Q1-Q3) [b]	184.2 (100.3 - 290.5)	190.4 (105.4 - 311.2)
Vegetable intake, g/day, median (Q1-Q3) [b]	155.8 (101.7 – 234.0)	156.8 (100.5 - 240.1)
Dairy intake, g/day, median (Q1-Q3) [b]	281.5 (150.0 - 458.3)	298.9 (168.2 - 468.4)
Fish and shellfish, g/day, median (Q1-Q3) [b]	28.2 (15.2 - 50.3)	29.7 (14.8 - 51.6)
Resistin concentrations, ng/ml, mean (SD) [b]	4.7 (2.0)	4.7 (2.2)
Adiponectin, µg/ml, median (Q1-Q3) [b]	6.6 (4.7-9.2)	7.2 (5.2-9.6)
HMW Adiponectin, µg/ml (Q1-Q3) [b]	3.4 (2.1-5.1)	3.7 (2.4-5.4)
Leptin, ng/ml (Q1-Q3) [b]	8.9 (4.6-17.5)	7.6 (3.7-16.8)
Soluble leptin receptor, ng/ml, mean (SD) [b]	21.4 (7.2)	22.8 (10.7)
Reactive oxygen metabolites, Carratelli units, mean (SD) [b]	399.5 (74.8)	384.9 (69.6)
CRP-hs, mgL, median (Q1-Q3) [b]	2.7 (1.0 - 4.8)	2.2 (0.9 - 4.4)
C-peptide, ng/ml, mean (SD) [b]	4.8 (2.7)	4.7 (2.8)
HDL-C, mmol/l, median (Q1-Q3) [b]	1.4 (1.2-1.7)	1.4 (1.2-1.8)
HbA1c, NGSP standardisation, mean (SD) [b]	5.9 (0.8)	5.8 (0.6)

a. Matching variable. b. Among users only.

Abbreviations: Q1-Q3, 25th–75th percentiles; SD, standard deviation; BMI, Body mass index; HMW, high-molecular weight; ROM, Reactive oxygen metabolites; HDL-C, High-density lipoprotein cholesterol (HDL-C); hsCRP, High-sensitivity C-reactive protein; HbA1c, glycated hemoglobin A1c.

Table S2. Association of resistin concentrations and risk of colorectal cancer in subgroups analyses.

	Quartile form					Continuous form	
	Q1	Q2	Q3	Q4	p trend [a]	Doubling resistin concentrations [b]	p value
Resistin quartile ranges (ng/ml)	≤3.47	3.47 < to ≤4.28	4.28 < to ≤5.42	5.42 < to ≤34.41			
By BMI [c d]							
BMI≥25							
No. cases/ controls	197/198	213/206	206/203	218/192		834/799	
RR (95%CI)	ref	1.05 (0.79 - 1.41)	1.03 (0.77 - 1.38)	1.19 (0.89 - 1.61)	0.65	1.17 (0.95 - 1.44)	0.13
BMI<25							
No. cases/ controls	100/126	104/119	142/118	113/131		459/494	
RR (95%CI)	ref	1.08 (0.73 - 1.61)	1.56 (1.05 - 2.30)	1.13 (0.76 - 1.70)	0.11	1.06 (0.81 - 1.39)	0.68
By hsCRP [c e]							
CRP ≥3mg/l							
No. cases/ controls	71/63	80/59	115/74	124/89		390/285	
RR (95%CI)	ref	1.16 (0.68 - 1.98)	1.47 (0.89 - 2.44)	1.45 (0.87 - 2.41)	0.38	1.31 (0.94 - 1.81)	0.11
CRP <3mg/l							
No. cases/ controls	134/122	125/124	122/106	97/90		478/442	
RR (95%CI)	ref	0.94 (0.64 - 1.37)	1.01 (0.68 - 1.50)	0.94 (0.62 - 1.43)	0.97	0.94 (0.70 - 1.27)	0.70
By length of follow-up [f]							
≤ 2 years							
No. cases/ controls	41/71	66/53	72/47	65/73		244/244	
RR (95%CI)	Ref	3.36 (1.71 - 6.62)	4.42 (2.25 - 8.71)	1.97 (1.06 - 3.64)	<0.001	1.44 (0.97 - 2.12)	0.07
2 years to ≤ 5 years							
No. cases/ controls	103/103	113/127	139/137	115/103		470/470	
RR (95%CI)	Ref	0.83 (0.56 - 1.24)	1.03 (0.70 - 1.54)	1.13 (0.74 - 1.73)	0.46	1.06 (0.80 - 1.42)	0.67
>5 years							
No. cases/ controls	153/150	138/145	137/137	151/147		579/579	
RR (95%CI)	ref	1.02 (0.73 - 1.44)	0.97 (0.69 - 1.37)	0.98 (0.69 - 1.39)	0.99	1.01 (0.79 - 1.29)	0.94
>2 years							
No. cases/ controls	256/253	251/272	276/274	266/250		1049/1049	
RR (95%CI)	ref	0.92 (0.72 - 1.19)	0.98 (0.76 - 1.27)	1.05 (0.81 - 1.37)	0.79	1.03 (0.86 - 1.24)	0.72
Complete case analysis (including only participants with no missing data on any variable)[f]							
No. cases/ controls, Model 1	276/290	280/297	315/285	288/285		1159/1157	

		RR (95%CI)	ref	1 (0.79 - 1.28)	1.19 (0.93 - 1.51)	1.08 (0.84 - 1.38)	0.46	1.12 (0.95 - 1.34)	0.18
Excluding participants with extreme resistin levels (from 8.38 to 34.41 ng/mL)									
		No. cases/ controls	292/310	304/314	336/307	262/263		1194/1194	
		RR (95%CI)	ref	1.06 (0.84 - 1.34)	1.19 (0.94 - 1.51)	1.08 (0.84 - 1.39)	0.51	1.10 (0.91 - 1.34)	0.32
By fasting status [c, d]									
	<=6 hours								
		No. cases/ controls	228/226	212/227	249/247	254/243		943/943	
		RR (95%CI)	ref	1.00 (0.77 - 1.31)	1.04 (0.79 - 1.36)	1.12 (0.84 - 1.47)	0.85	1.04 (0.86 - 1.25)	0.70
	>6 hours								
		No. cases/ controls	69/98	105/98	99/74	77/80		350/350	
		RR (95%CI)	ref	**1.76 (1.11 - 2.79)**	**2.09 (1.30 - 3.34)**	**1.47 (0.91 - 2.36)**	**0.02**	**1.45 (1.03 - 2.03)**	**0.03**
By colon cancer categorizing [f]									
	Distal colon cancer								
		No. cases/ controls	86/92	109/107	99/100	108/103		402/402	
		RR (95%CI)	ref	1.14 (0.74 - 1.75)	1.18 (0.75 - 1.85)	1.23 (0.80 - 1.9)	0.82	1.14 (0.85 - 1.54)	0.39
	Proximal colon cancer								
		No. cases/ controls	77/80	69/85	100/87	103/97		349/349	
		RR (95%CI)	ref	0.95 (0.59 - 1.52)	1.24 (0.77 - 2.02)	1.2 (0.73 - 1.97)	0.65	1.24 (0.89 - 1.72)	0.20

a. P values for trend derived from models with the median resistin concentration within quartiles as a continuous variable.
b. Models with continuous log-transformed resistin concentrations by log 2.
c. Results were based on unconditional logistic regression models adjusted for age, sex (men, women with classifying by menopausal status, phase of the menstrual cycle at blood collection and use of oral contraceptives at blood collection; and hormone replacement therapy use in postmenopausal women), study center, time of the day at blood collection, and fasting status, smoking status, education, alcohol consumption, alcohol abstainers, physical activity index, energy intake, red meat, processed meat, dietary fiber, fruit intake, vegetable intake, dairy intake, fish and shellfish intake, BMI, and residuals of BMI-adjusted waist circumference.
d. BMI and residuals of BMI-adjusted waist circumference were not included in the model as a covariate.
e. Data for hsCRP were only available on 868 cases and 727 controls.
f. Results were based on conditional logistic regression models conditioned on matching factors (age, sex, study center, time of the day at blood collection, and fasting status; women were further matched by menopausal status, phase of the menstrual cycle and use of oral contraceptives at blood collection; postmenopausal women were matched by hormone replacement therapy use) and adjusted for smoking status, education, alcohol consumption, alcohol abstainers, physical activity index, energy intake, red meat, processed meat, dietary fiber, fruit intake, vegetable intake, dairy intake, fish and shellfish intake, body mass index (BMI), and residuals of BMI-adjusted waist circumference.

Table S3. Characteristics of the studies investigated the relationship between circulating resistin concentrations and risk of colorectal cancer

	Author, Year	Study design	Country	Blood sample	No. of participants	Assay kit	Assay manufacturer	Pre-diagnostic resistin	ORs/RRs (95%CI) [a] resistin levels were used as continuous variables; [b] log-transformed resistin levels	Covariates
1	Mihajlovic M 2019	Case-control	Serbia	Plasma	CRC 86/ 75 control	ELISA	R&D Systems	No	**[a]1.06 (1.01–1.10) P< 0.05 Per 1 unit of Resistin (ng/mL)**	age, gender, BMI, HDLC, and LDL-C
2	Joshi et al. 2014	Case-control	South Korea	Serum	CRC 100/ 100 control	ELISA	Adipogen	No	**Low (<median): Ref**<br>**High (≥median): 5.44 (2.97-9.97) P< 0.001. Median not shown**	Age, sex-matched odds ratio
3	Danese et al., 2012	Case-control	Italy	Serum	CRC 40/ 40 control	ELISA	Mediagnost	No	**[ab]1.33 (1.03-1.72) per SD log resistin, P = 0.020**	Age, sex, BMI, and lifestyle parameters (smoking status, alcohol consumption, physical activity, family history of CRC and for women menopausal status and hormone replacement therapy)
4	Nakajima et al., 2010	Case-control	Japan	Plasma	CRC 115/115 control	ELISA	BioVendor	No	**[ab]2.07 (1.05–4.06), p=0.03) per SD log resistin,**	Used age-, sex, and BMI-matched control.
5	Otake et al., 2010	Case-control	Japan	Plasma	CRC 51/26 controls	ELISA	BioVendor	No	Low (< 42 ng/mL): Ref<br>High (as ≥ 42 ng/mL), early cancer: 0.980 (0.32-2.81) p=0.969 ;<br>High (as ≥ 42 ng/mL), advanced cancer: 0.67 (0.19-2.33) p=0.53	No covariate included.
6	Ho GY et al., 2012	Case-cohort	USA (WHI-OS)	Plasma	CRC 456/ 834 healthy people	Multiplex immunoassay	Millipore	Yes	Q1 (<10ng/mL): Ref<br>Q2 (10.0–12.5): 0.84 (0.57–1.24)<br>Q3 (12.6–15.7): 0.90 (0.62–1.32)<br>Q4 (≥15.8): 1.04 (0.72–1.50)<br>P=0.73	Age, race, smoking, history of colonoscopy, waist circumference, and estrogen level
